# Immunological Landscapes in Lung Transplantation: Insights from T Cell Profiling in BAL and PBMC

**DOI:** 10.3390/ijms25052476

**Published:** 2024-02-20

**Authors:** Tharushi Ayanthika de Silva, Simon Apte, Joanne Voisey, Kirsten Spann, Maxine Tan, Daniel Chambers, Brendan O’Sullivan

**Affiliations:** 1 Centre for Genomics and Personalised Health, Faculty of Health, School of Biomedical Sciences, Queensland University of Technology (QUT), Brisbane, QLD 4001, Australia; t.desilva@qut.edu.au (T.A.d.S.);; 2 Queensland Lung Transplant Service, Ground Floor, Clinical Sciences Building, The Prince Charles Hospital, Brisbane, QLD 4001, Australia; 3 Facility of Clinical Medicine, The University of Queensland, Brisbane, QLD 4001, Australia; 4 Centre for Immunology and Infection Control, Faculty of Health, School of Biomedical Sciences, Queensland University of Technology (QUT), Brisbane, QLD 4001, Australia; kirsten.spann@qut.edu.au

**Keywords:** lung transplant, CLAD, Tregs, granzyme B, BAL, PBMC

## Abstract

Lung transplant recipients frequently encounter immune-related complications, including chronic lung allograft dysfunction (CLAD). Monitoring immune cells within the lung microenvironment is pivotal for optimizing post-transplant outcomes. This study examined the proportion of T cell subsets in paired bronchoalveolar lavage (BAL) and peripheral PBMC comparing healthy (*n* = 4) and lung transplantation patients (*n* = 6, no CLAD and *n* = 14 CLAD) using 14-color flow cytometry. CD4+ T cell proportions were reduced in CD3 cells in both PBMC and BAL, and positive correlations were discerned between T cell populations in peripheral PBMC and BAL, suggesting the prospect of employing less invasive PBMC sampling as a means of monitoring lung T cells. Furthermore, regulatory T cells (Tregs) were enriched in BAL when compared to peripheral PBMC for transplant recipients. A parallel positive correlation emerged between Treg proportions in BAL and peripheral PBMC, underscoring potential avenues for monitoring lung Tregs. Finally, the most promising biomarker was the Teff (CD8+Granzyme B+)–Treg ratio, which was higher in both the PBMC and BAL of transplant recipients compared to healthy individuals, and increased in the patients with CLAD compared to no CLAD and healthy patients. Conclusions: Distinct T cell profiles in BAL and peripheral PBMC underscore the significance of localized immune monitoring in lung transplantation. The Teff (CD8+granzyme B+)–Treg ratio, particularly within the context of CLAD, emerges as a promising blood and BAL biomarker reflective of inflammation and transplant-related complications. These findings emphasize the imperative need for personalized immune monitoring strategies that tailored to address the unique immunological milieu in post-transplant lungs.

## 1. Introduction

Lung transplantation is a treatment option for end-stage lung disease. Lung transplant recipients require immunosuppressive drugs to manage chronic rejection, and these have undesirable side effects, including malignancy and infections (reviewed in [1]). After transplantation, damage caused by the immune system to the lung allograft can result in rejection, which can eventually lead to chronic lung allograft rejection (CLAD) (reviewed in [2]).

CLAD has been shown to affect 50% of lung transplant recipients by five years after transplant [3], and it remains the leading cause of mortality in lung transplant recipients [4,5].

With several studies examining animal models of heart, lung, and liver transplantation discovering that regulatory T cells (Tregs) are beneficial in allograft survival (reviewed in [6]), knowledge about possible ways to utilize Tregs to promote tolerance in lung transplantation is increasing.

Although many studies have investigated the role and association of Tregs in CLAD-free survival after lung transplantation, they report inconsistent data. Some studies show an increase in peripheral Tregs in CLAD-free patients compared with those who developed CLAD [7,8,9,10]. In contrast, some studies show that an increase in circulating Tregs is associated with the development of CLAD [11,12]. Furthermore, characterization of T cell populations is highly variable across studies, highlighting the need for detailed investigation of T cell phenotypes in lung transplant recipients.

Although most studies have focused on circulating Tregs, only a handful of studies have looked at Tregs in the lung utilizing bronchoalveolar lavage (BAL) fluid [8,13,14] or transbronchial biopsy tissue [15,16,17,18]. And while most studies show that increased Tregs in BAL is associated with rejection or CLAD [8,13,18], two groups show no change in Tregs associated with CLAD [14,16,17].

Bronchoalveolar lavage (BAL) is a standard technique in respiratory medicine for investigating airways and alveolar space. In a lung transplant setting, BAL is part of the routine surveillance bronchoscopy procedure performed to detect rejection and infections within the lung [19]. BAL is considered safer and less invasive than TBB, providing a unique insight into the lung microenvironment of a larger area of the lung [20]. TBB yields all cells in the tissue, but BAL washes out the cells in alveolar space, which consists mainly of alveolar macrophages and minor populations of lymphocytes and granulocytes (reviewed in [21]). Characterizing cells collected via a BAL enables us to describe the phenotype of cells implicated in allo-immune responses.

Investigating both BAL and PBMC simultaneously collected from patients can provide a unique opportunity to understand both local and systemic changes in cells occurring due to transplantation. Hence, it was hypothesized that the T cell profile in the lung would be different compared to that of the PBMC, and this was investigated by comparing T cells in BAL in contrast with T cells in peripheral PBMC collected simultaneously. This study serves as a proof-of-concept study to explore both the regulatory and inflammatory arms of the immune system in BAL and PBMC using multicolour flow-cytometry. Understanding the nature and balance between Tregs and inflammatory cells will give insight into the underlying mechanisms of rejection and tolerance. This study investigated the phenotype of T cells based on markers expressed in populations of regulatory T cells (Tregs), memory T cells (Tmem) and effector T cells (Teff), which were identified in peripheral PBMC and BAL using flow-cytometry.

## 2. Results

### 2.1. Multi-Colour Flow Cytometric Panel to Identify T Cell Subsets Comparing PBMC and BAL

**Figure 1:**
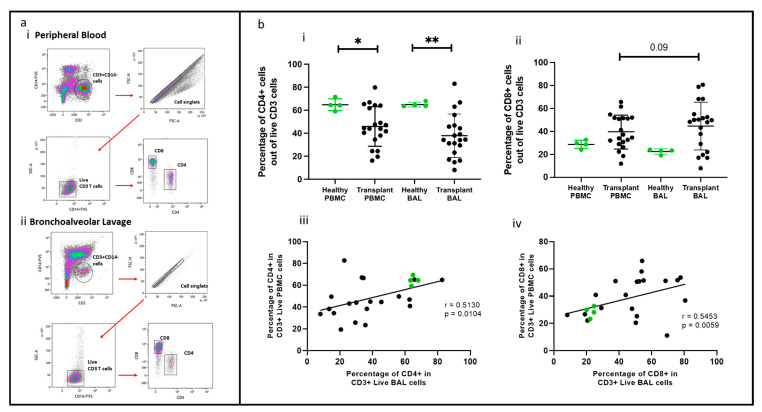
T cell populations in BAL and blood. (**a**) Flow cytometry plots depicting identified T cell populations in peripheral blood and bronchoalveolar lavage. (**b**) (**i**) CD4+ T cells (**ii**) CD8+ T cells. (**b**) (**iii**) Cell proportions of CD4+ T cells between blood and BAL. (**b**) (**iv**) Cell proportions of CD8+ T cells between blood and BAL * *p* ≤ 0.05; ** *p* ≤ 0.01.

A 14-color flow cytometric panel was developed to identify various T lymphocyte subsets, including regulatory T cells (Tregs) marked by CD25, CD127, CD39, and CD73, as well as FoxP3 and effector T cells (Teff) identified through granzyme B expression, proliferating cells indicated by Ki67, and memory and migratory cells differentiated by CD45RO and CD194, respectively. The optimal working concentrations for these markers were determined through optimization, and the validity of positive populations was confirmed using ‘Fluorescence Minus One’ (FMO) controls. In initial experiments, we devised a gating strategy to distinguish lymphocytes in bronchoalveolar lavage (BAL) from other cell types, particularly alveolar macrophages, which are auto fluorescent and tend to bind antibodies non-specifically. This strategy involved combining a viability stain (FVS 620) and CD14 on a single channel, gating on FVS-CD14-CD3+ cells, excluding doublets, and then gating on CD4 or CD8 (Figure 1a(i,ii)). Flow cytometry analysis of paired PBMC and BAL samples from a lung transplant recipient with chronic lung allograft dysfunction (CLAD) demonstrated the panel’s capability to identify CD4 and CD8 T cells in both PBMC and BAL. There was no difference in using this gating strategy, and proportions of T cell subsets were compared for PBMC and BAL samples from 20 transplant recipients and four healthy individuals.

### 2.2. CD4 and CD8 T Cells Comparing PBMC and BAL

CD4+ and CD8+ T cells were identified using the gating strategy depicted in Figure 1. CD4+ cells were more abundant in the healthy cohort than in the transplant cohort in PBMC (*p* = 0.045) and BAL (*p* = 0.007). Conversely, CD8+ T cell proportions showed no significant difference between PBMC and BAL in either cohort (Figure 1b(i,ii)). Proportions of CD4+ and CD8+ T cells among CD3+ cells in PBMC correlated with their counterparts in BAL (r = 0.513, *p* = 0.010 and r = 0.545, *p* = 0.006) (Figure 1b(iii,iv)). We next compared absolute PBMC CD3+CD4+ cell numbers for healthy (*n* = 4, median = 252 ± 121) cells/μL of blood) and the transplant cohort (*n* = 20, median = 391 ± 416) cells/μL of blood and found no differences (Mann–Whitney *U* Test, *p* = 0.851). Similarly, absolute PBMC CD3+CD8+ cell numbers for healthy (*n* = 4, median = 116, SD= 72) cells/μL of blood) and the transplant cohort (*n* = 20, median= 391, SD= 416) cells/uL of blood were similar (Mann–Whitney *U* Test, *p* = 0.114). Given the absence of substantial variances in T cell quantities in the bloodstream between the healthy and transplant groups, alongside challenges in acquiring absolute counts from bronchoalveolar lavage, comparisons were conducted based on cellular proportions in subsequent analyses.

### 2.3. Effector T Cells

**Figure 2:**
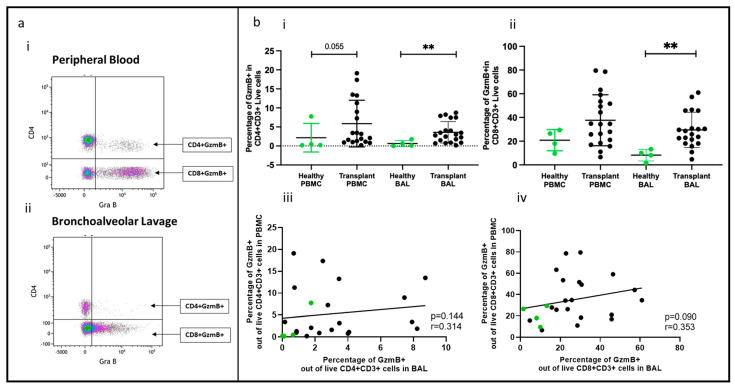
Effector T cells in BAL and peripheral blood. (**a**) Flow cytometry plots depicting effector T cell populations (expressing granzyme B) in (**i**) peripheral blood and (**ii**) bronchoalveolar lavage. (**b**) (**i**) CD4+GZMB+ T cells; (**ii**) CD8+GZMB+ T cells; (**iii**) cell proportions of CD4+GZMB+ T cells between blood and BAL; (**iv**) cell proportions of CD8+GZMB+ T cells between blood and BAL. ** *p* ≤ 0.01.

Effector T cells expressing granzyme B, a crucial enzyme involved in acute rejection mediated by T cells, were identified, as shown in Figure 2a(i,ii). The proportions of CD4+granzyme B+ T cells and CD8+granzyme B+ T cells (out of CD4+ and CD8+ cells, respectively) were higher in the BAL of transplant recipients than in healthy controls (*p* = 0.0072, *p* = 0.0023), but no significant difference was observed in PBMC between the two groups (transplant vs. healthy) (Figure 2b(i,ii)). Notably, there was no correlation between the proportions of CD4+ or CD8+ cells in PBMC and their counterparts in BAL (Figure 2b(iii,iv)).

### 2.4. Memory T Cell Populations Comparing PBMC and BAL

**Figure 3:**
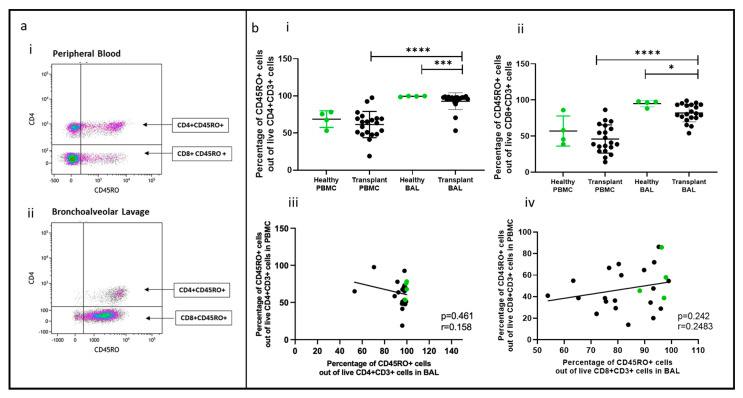
Memory T cells in BAL and peripheral blood. (**a**) Flow cytometry plots depicting memory T cell populations (expressing CD45RO) in (**i**) peripheral blood and (**ii**) bronchoalveolar lavage. (**b**) (**i**) CD4+CD45RO+ T cells (**ii**) CD8+CD45RO+ T cells. (**iii**) Cell proportions of CD4+CD45RO+ T cells between blood and BAL; (**iv**) cell proportions of CD8+CD45RO+ T cells between blood and BAL. * *p* ≤ 0.05; *** *p* ≤ 0.001; **** *p* ≤ 0.0001.

Memory T cell populations in PBMC and BAL were analyzed based on CD45RO expression, as shown in Figure 3a(i,ii). Proportions of CD45RO+CD4+ and CD45RO+CD8+ T cells were higher in transplant BAL than in PBMC (*p* < 0.0001, *p* = 0.0008). In comparison to healthy participants, memory CD4+ and CD8+ T cells were reduced in transplant BAL (*p* = 0.0125, *p* = 0.023) (Figure 3b(i,ii)). No significant difference was observed between healthy BAL and PBMC for either CD4+ or CD8+ memory T cells, and there was no correlation between memory cell proportions in PBMC and paired BAL (Figure 2b(iii,iv)).

### 2.5. Non-Tregs Expressing CD39 Comparing PBMC and BAL

**Figure 4:**
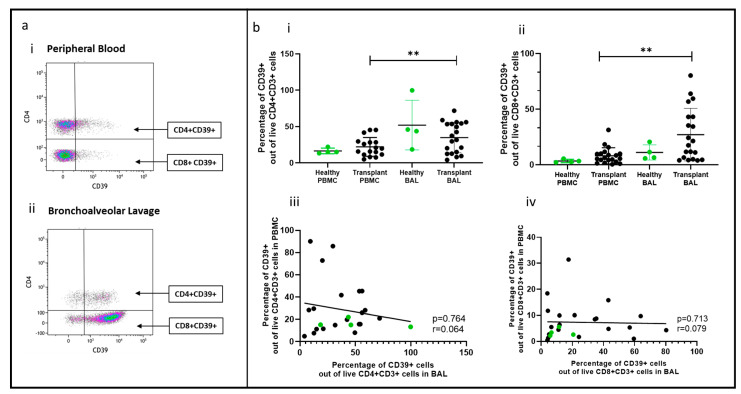
Non-Tregs expressing CD39 in BAL and peripheral blood. (**a**) Flow cytometry plots depicting non-Treg population expressing CD39 cell populations in (**i**) peripheral blood and (**ii**) bronchoalveolar lavage. (**b**) (**i**) CD4+CD39+ T cells (**ii**) CD8+CD39+ T cells; (**iii**) cell proportions of CD4+CD39+ T cells between blood and BAL; (**iv**) cell proportions of CD8+CD39+ T cells between blood and BAL. ** *p* ≤ 0.01.

A novel population of CD4+ and CD8+ non-Treg cells expressing CD39 was identified in BAL (Figure 4a(i,ii)), and their proportions were higher than in PBMC (*p* = 0.0093, *p* = 0.0032) (Figure 4b(i,ii)). This population was also found in healthy control BAL, with similar CD39+ cell proportions in transplant recipients and healthy samples. However, there was no correlation between CD4+ or CD8+ CD39+ cell proportions in PBMC and paired BAL (Figure 4b(iii,iv)).

### 2.6. Regulatory T Cells Comparing PBMC and BAL

**Figure 5:**
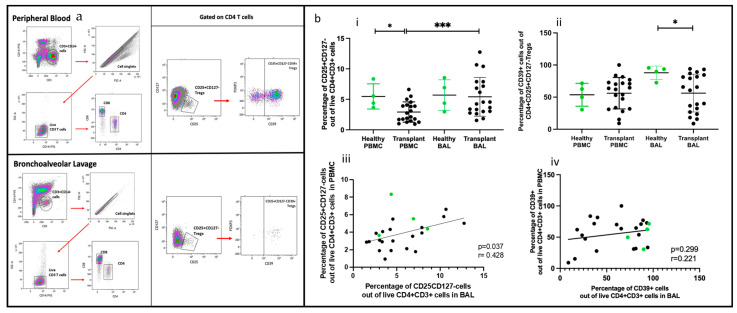
Regulatory T cells in BAL and peripheral blood. (**a**) Flow cytometry plots depicting conventional Tregs (CD25+CD127−) and Tregs expressing CD39 cell populations in (**i**) peripheral blood and (**ii**) bronchoalveolar lavage. (**b**) (**i**) CD4+CD25+CD127− Tregs (**ii**) CD4+CD25+CD127−CD39+ T cells; (**iii**) cell proportions of CD4+CD25+CD127− T cells between blood and BAL; (**iv**) cell proportions of CD4+CD25+CD127−CD39+ T cells between blood and BAL.* *p* ≤ 0.05; *** *p* ≤ 0.001.

Treg subsets in PBMC and BAL were identified using CD4+CD25+CD127−. For Treg validation, FoxP3 was included, along with CD39 as a marker of Treg activation (Figure 5a(i,ii)). FOXP3 was unreliable for identifying Tregs in BAL, as CD4+CD25+CD127− cells were sometimes negative for FOXP3. Therefore, CD25+CD127− was used to define Tregs, with CD39 serving as a marker of activation (Figure 5a(i,ii)).

Of the two populations, Tregs (CD4+CD25+CD127−) were more abundant in transplant BAL than in PBMC (*p* = 0.0006) (Figure 5b(i,ii)). Activated Tregs (CD25+CD127−CD39+) proportions were similar in BAL and PBMC, but they were higher in the BAL of healthy participants than in the transplant group (*p* = 0.023). Paired PBMC and BAL CD4+CD25+CD127− Tregs showed a positive correlation (*p* = 0.037, r = 0.428), but not CD4+ CD25+CD127−CD39+ Tregs (Figure 5b(iii,iv)).

### 2.7. Subpopulation of Tregs Comparing PBMC and BAL

**Figure 6:**
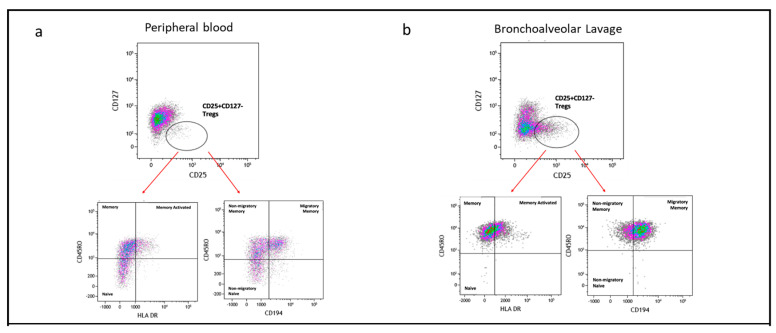

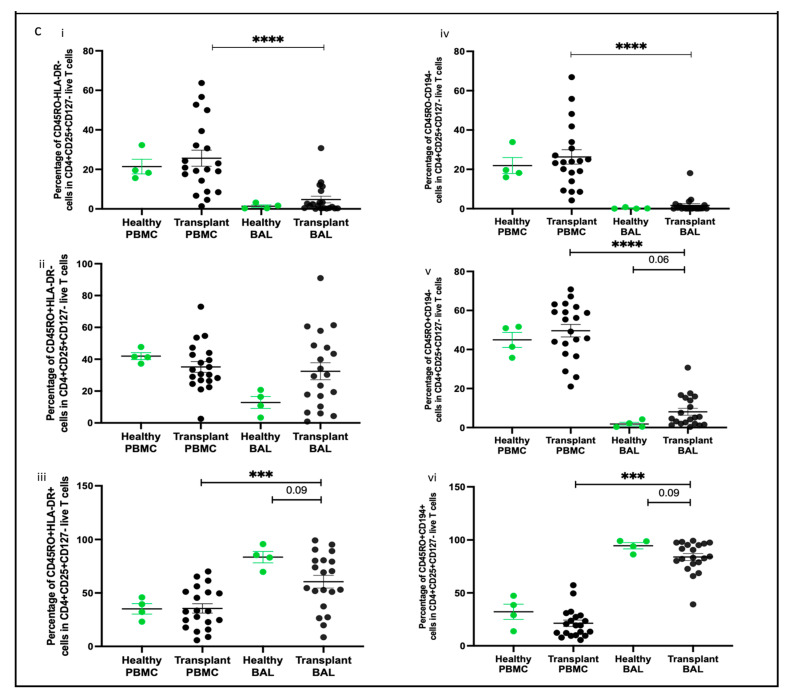
Subpopulation of Tregs based on CD45RO, HLA-DR, and CD194. (**a**) Flow cytometry plots depicting Treg subpopulations in peripheral blood; (**b**) flow cytometry plots depicting Treg subpopulations in bronchoalveolar lavage. (**c**) (**i**) naïve Tregs (CD45RO−HLA-DR−); (**ii**) memory Tregs (CD45RO+HLA-DR−); (**iii**) memory activated Tregs (CD45RO+HLA-DR+); (**iv**) non-migratory naïve Tregs (CD45RO−CD194−); (**v**) non-migratory memory Tregs (CD45RO+CD194−); (**vi**) migratory memory Tregs (CD45RO+CD194+). *** *p* ≤ 0.001; **** *p* ≤ 0.0001.

We further classified Tregs (CD4+CD25+CD127−) into subpopulations based on the expression of the CD45RO memory marker, HLA-DR (indicative of antigen experience), and CD194 (associated with migratory and tissue-resident characteristics). Representative flow cytometry plots illustrating these Treg subpopulations in peripheral PBMC mononuclear cells (PBMC) and bronchoalveolar lavage (BAL) are presented in Figure 6a,b.

By assessing CD45RO and HLA-DR expression, we identified three subpopulations of Tregs: naïve Tregs (CD45RO−HLA-DR−), memory Tregs (CD45RO+HLA-DR−), and memory activated Tregs (CD45RO+HLA-DR+). Naïve Treg proportions were higher in PBMC compared to BAL (*p* = 0.0004) (Figure 6c(i)), while memory Treg proportions were similar between the two compartments (Figure 6c(ii)). Memory activated Treg proportions were higher in BAL than in PBMC (*p* = 0.0212) (Figure 6c(iii)). Interestingly, no significant correlations were observed between the subpopulations of Tregs based on CD45RO and HLA-DR expression in BAL and their counterparts in PBMC.

We also analysed Treg subpopulations based on CD45RO and CD194 expression, identifying three categories: naïve Tregs (CD45RO−CD194−), memory Tregs (CD45RO+CD194−), and migratory memory Tregs (CD45RO+CD194+). Naïve Treg proportions were greater in PBMC compared to BAL (*p* = 0.0016, *p* < 0.0001) (Figure 6c(iv,v)). Both memory Treg and migratory memory Treg proportions were higher in BAL than in PBMC (*p* < 0.0001) (Figure 6c(vi)).

### 2.8. Teff and Treg Balance (Granzyme B:Treg Ratio) Comparing PBMC and BAL

**Figure 7:**
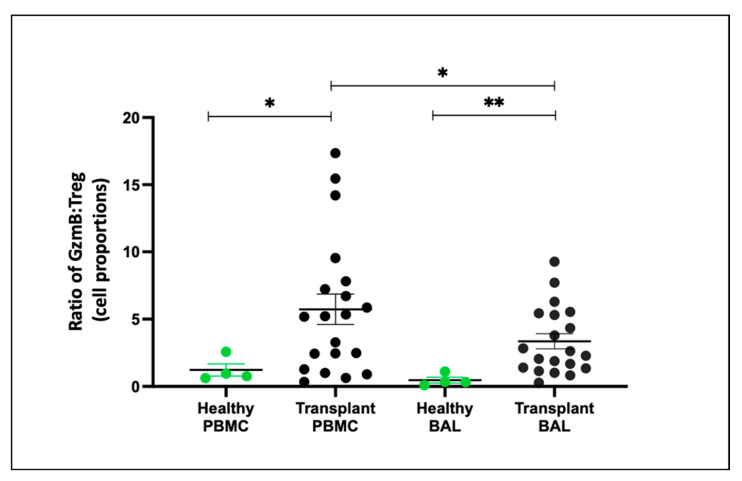
Granzyme B–Treg Ratio Between Healthy and Transplant (GZMB- Granzyme B). The ratio of CD8+GZMB+ T cell proportion to CD4+CD25+CD127− Treg proportion between healthy and transplant cohorts in BAL and PBMC. * *p* ≤ 0.05; ** *p* ≤ 0.01.

To assess the balance between effector T cells (Teffs) and regulatory T cells (Tregs), we examined the ratio of Tregs (CD4+CD25+CD127−) to Teffs (CD8+granzyme B+) proportions. In both PBMC and BAL, transplant recipients exhibited a higher granzyme B:Treg ratio compared to healthy individuals (*p* = 0.045, *p* = 0.003) (Figure 7). Notably, BAL samples from transplant recipients had a reduced granzyme B–Treg ratio compared to paired PBMC samples (*p* = 0.012).

### 2.9. T Cell Subsets and Association with CLAD

#### 2.9.1. Effector T Cells (Teffs) and Memory T Cells (Tmems) Comparing PBMC and BAL

**Figure 8:**
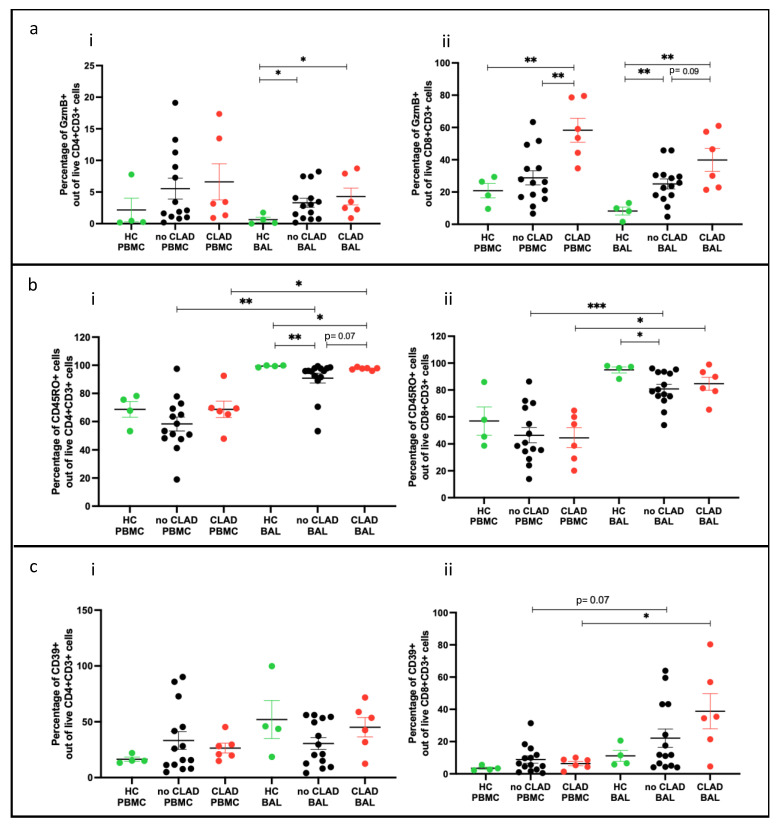
T cell subtypes comparing groups. (**a**) Effector T cells across groups (**i**) of CD4+GZMB+ cells; (**ii**) of CD8+GZMB+ cells. (**b**) Memory T cells across groups (**i**) of CD4+CD45RO+ cells; (**ii**) of CD8+CD45RO+ cells. (**c**) CD39 expressing cells across groups (**i**) of CD4+CD39+ cells; (**ii**) of CD8+CD39+ cells. * *p* ≤ 0.05; ** *p* ≤ 0.01; *** *p* ≤ 0.001.

We examined effector T cells (Teffs) and memory T cells (Tmems) in participants stratified into three groups: ‘healthy control’ (HC: *n* = 4), ‘no CLAD’ (unaffected by CLAD: *n* = 14), and ‘CLAD’ (affected by CLAD: *n* = 6), comparing differences in cell proportions. In BAL, proportions of CD4+ and CD8+ Teffs (granzyme B+) were higher in both CLAD (*p* = 0.019, *p* = 0.0095) and no CLAD (*p* = 0.018, *p* = 0.0078) groups compared to the healthy group (Figure 8a(i,ii)). In PBMC, proportions of CD8+ Teffs were higher in the CLAD group than in the healthy group (*p* = 0.0046) (Figure 8a(ii)).

Memory CD4+CD45RO+ T cells were lower in both CLAD and no CLAD BAL than in the healthy group (*p* = 0.0016). Memory CD8+CD45RO+ T cells were lower in no CLAD BAL compared to the healthy group (*p* = 0.013). Comparing PBMC and BAL, both CLAD and no CLAD groups had higher proportions of CD4+ and CD8+ memory T cells in BAL (Figure 8b(ii)).

In addition, CD8+CD39+ T cells were more abundant in CLAD BAL than in PBMC (*p* = 0.031). A similar trend was observed between no CLAD BAL and PBMC, although it did not reach statistical significance (Figure 8c(ii)).

#### 2.9.2. Treg Populations Comparing PBMC and BAL

**Figure 9:**
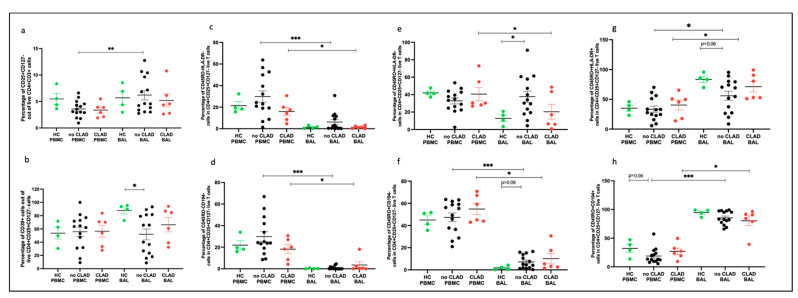
Tregs comparing groups. (**a**) Conventional Tregs (CD4+CD25+CD127−); (**b**) CD39+ Tregs; (**c**) naïve Tregs (CD45RO+HLA-DR+); (**d**) non-migratory naïve Tregs (CD45RO−CD194−); (**e**) memory Tregs (CD45RO+HLA-DR−); (**f**) non-migratory memory Tregs (CD45RO+CD194−); (**g**) memory activated Tregs (CD45RO+HLA-DR+); (**h**) migratory memory Tregs (CD45RO+CD194+). * *p* ≤ 0.05; ** *p* ≤ 0.01; *** *p* ≤ 0.001.

Comparing PBMC and BAL, it was observed that, in the no CLAD group, BAL had a higher proportion of CD4+CD25+CD127− T cells than PBMCs (*p* = 0.0052) (Figure 9a). Moreover, the no CLAD group exhibited a lower proportion of activated Tregs (CD25+CD127−CD39+) compared to the healthy control (HC) group (*p* = 0.018) (Figure 9b).

In terms of Treg subpopulations, both the no CLAD (*p* = 0.0009) and CLAD (*p* = 0.031) groups displayed a higher proportion of naïve Tregs (CD45RO−HLA-DR−) in PBMCs than in BAL (Figure 9c). Similarly, non-migratory Tregs (CD45RO−CD194−) were more abundant in PBMCs than in BAL for both the no CLAD (*p* = 0.0001) and CLAD (*p* = 0.031) groups (Figure 9d).

Memory Tregs (CD45RO+HLA-DR−) were more prevalent in no CLAD BAL than in HC BAL (*p* = 0.035). Interestingly, CLAD PBMCs exhibited the highest proportion of memory Tregs in relation to BAL (*p* = 0.031) (Figure 9e). Moreover, CLAD PBMCs also had a higher proportion of non-migratory memory Tregs (CD45RO+CD194−) compared to BAL (*p* = 0.031). Likewise, no CLAD PBMCs showed a higher proportion of non-migratory memory cells than BAL (*p* = 0.0001) (Figure 9f).

Memory activated Tregs (CD45RO+HLA-DR+) were more abundant in the BAL of both the no CLAD (*p* = 0.020) and CLAD (*p* = 0.031) groups than in paired PBMC. Although no CLAD BAL had a lower proportion of memory activated Tregs compared to HC BAL, this difference was not statistically significant (Figure 9g). Additionally, migratory memory Tregs (CD45RO+CD194+) were more prevalent in the BAL of both the no CLAD and CLAD groups (*p* = 0.031) than in the paired PBMCs (*p* = 0.0001) (Figure 9h).

#### 2.9.3. Teffs and Tregs Balance (Granzyme B–Treg Ratio) Comparing PBMC and BAL

**Figure 10:**
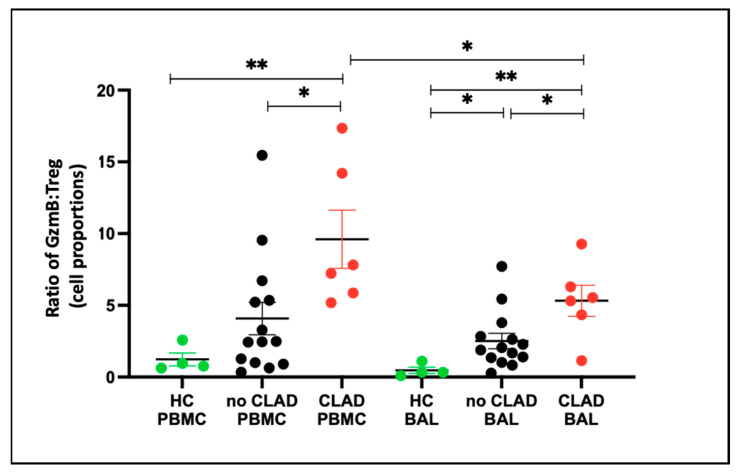
Granzyme B–Treg ratio between healthy and transplant (GZMB- granzyme B). Ratio of CD8+GZMB+ T cell proportion: CD4+CD25+CD127− Treg Proportion across groups in PBMC and BAL. * *p* ≤ 0.05; ** *p* ≤ 0.01.

To gain insight into the balance of granzyme B and Treg cells under different conditions, we evaluated the granzyme B–Treg ratio for each group. In PBMCs, the granzyme B–Treg ratio was higher in the CLAD group compared to the healthy control (HC) group (*p* = 0.0095) and lower in the no CLAD group than in the CLAD group (*p* = 0.015). Conversely, in BAL, the granzyme B–Treg ratio was higher in both the CLAD (*p* = 0.0001) and no CLAD (*p* = 0.012) groups than in HC. Moreover, CLAD BAL exhibited a higher ratio than no CLAD BAL (*p* = 0.041) (Figure 10).

## 3. Discussion

In this study, we analysed 20 transplant recipients and 4 healthy participants. We examined immune cells from both bronchoalveolar lavage (BAL) and blood samples collected on the same day. We used a 14-color multi-parameter flow cytometric panel specifically designed to identify various subsets of T cells. It is worth noting that BAL cells exhibited high auto-fluorescence due to the presence of alveolar macrophages. We then isolated lymphocytes based on size and granularity, and further gated them based on CD3 expression, excluding dead cells and monocytes (CD14+), using a CD14 dump channel. Within the CD3 subset, we identified CD4+ and CD8+ T cells, categorizing them into effector (expressing granzyme B) and regulatory phenotypes (expressing CD25+CD127- and CD39). Both CD4+ and CD8+ T cells play important roles in the allorecognition of transplanted lungs by the recipient’s immune system, as reviewed in [22]. CD4+ T cells activate the immune response when exposed to foreign major histocompatibility complexes (MHCs) on the transplanted lungs, also enhancing the immune response through cytokine secretion [23]. In contrast, CD8+ T cells primarily act as cytotoxic T cells, secreting granzymes and perforin, which can lead to alloimmune damage in transplantation [24].

When comparing CD4+ and CD8+ T cells in transplant and healthy control (HC)- paired BAL and PBMC samples, we found that the proportions of these cells were similar. However, the proportions of CD4+ T cells in BAL and PBMC were lower in the transplant group compared to the HC group, although the absolute cell numbers in blood were similar. We also detected positive associations between blood and BAL for the main T cell populations of CD4+ and CD8+, suggesting that monitoring these cell populations in blood can provide insights into the composition of lung T cells via BAL.

Identifying FOXP3 in Tregs was challenging due to its downregulation and staining constraints in BAL cells. Therefore, we identified Tregs by their expression of CD4+CD25+CD127−, an approach commonly used in many studies to characterize and isolate FOXP3+ Tregs [25,26,27,28]. In the transplant groups, the proportion of Tregs was higher in BAL than in PBMC, indicating an enrichment of Tregs in CD4+ cells within the lung. Moreover, these cells were of a memory phenotype (CD45RO+, CD194+) indicating activation, which is consistent with published findings showing tissue-resident Tregs responding to inflammation or antigen stimulation by upregulating immunosuppressive molecules and homing receptors [29]. Tregs can be categorized as naïve, memory, and effector Tregs. Naïve Tregs have minimal suppressive function and little history of antigen exposure, while effector Tregs are activated upon antigen exposure with enhanced suppressive properties. Memory Tregs, on the other hand, are activated Tregs that can give rise to long-lived antigen-specific cells [29,30]. Both effector and memory Tregs play crucial roles in regulating tissue immunity due to their suppressive capacity, while naïve Tregs are often considered a reservoir for replenishment [31]. Tregs expressed CD39 in the BAL and PBMC of both healthy control and transplant recipients, with a lower proportion of CD25+CD127−CD39+ cells in the BAL of transplant recipients. CD39 is an ecto-enzyme that hydrolyzes ATP to immunosuppressive adenosine, limiting effector T cell proliferation and enhancing the suppressive function of Tregs [32,33]. Since CD39 may stabilize Tregs under inflammatory conditions [32,34], these results suggest that immunosuppressive protocols and/or inflammation in transplantation may perturb Treg function and limit their suppressor function. Importantly, the proportion of Tregs (CD25+CD127−) in BAL is positively correlated with PBMC, suggesting that less invasive blood collection and analysis could be used to monitor Tregs in the lung.

In addition to Tregs, a high proportion of CD4+ and CD8+ T cells in the lung were found to express CD39. The enrichment of these cells in the lung compared to peripheral blood suggests that they may play a crucial role in the lung microenvironment. Previous studies have identified T cells expressing CD39 in tumor microenvironments (reviewed in [35]) and inflammatory lesions, featuring two phenotypes of CD39+ memory and CD39+ Tregs [36]. Intriguingly, T cells expressing CD39 lack the ability to suppress T cell proliferation, unlike conventional Tregs [37]. Furthermore, CD39+CD8+ T cells have been shown to have an exhaustive phenotype [38]. This phenotype could pose a significant risk to lung health, potentially leading to damage and fibrosis. Exhausted T cells may fail to effectively combat infections, while elevated levels of CD39 could promote increased lung adenosine, a factor linked to conditions like idiopathic pulmonary fibrosis, asthma, and COPD [39]. The role of CD39+ non-Treg T cells remains unclear, warranting further investigation in the lung.

We identified effector T cells by their expression of granzyme B, a protease secreted by T cells in response to antigen stimulation. Previous studies have shown that granzyme B damages the alveolar space during alloimmune events in transplantation (reviewed in [40]). In our study cohort, both CD4+granzyme B+ and CD8+granzyme B+ T cells were higher in the BAL of the transplant groups compared to healthy controls. These cells were also antigen-experienced with a memory phenotype (CD45RO+) and enriched in the lung compared to PBMC. These findings suggest that, despite comprehensive immunosuppressive regimens, lung transplant recipients still experience ongoing alloimmune inflammation in the lungs. This underscores the importance of immune monitoring to assess the risk of lung damage.

To explore this concept further, we compared healthy controls with lung transplant recipients who had normal lung function (no chronic lung allograft dysfunction or CLAD) and those with reduced lung function (CLAD). We found that, in effector T cells and memory T cells, both CD4+ and CD8+ and were proportionally higher in the CLAD group compared to BAL in the healthy control group. The most significant differences were observed in the Teff (granzyme B+)–Treg ratio, which increased in the order of CLAD > no-CLAD > healthy controls in both blood and BAL. This ratio can be considered a valuable measure reflecting clear differences among the compared groups. The highest inflammation was detected in the CLAD group, as indicated by the high ratio, suggesting a potential role in the pathogenesis of CLAD. This measure may serve as an efficient biomarker reflecting the degree of inflammation within the lungs and in the systemic circulation.

We recognize certain limitations in our study, notably the small sample size of the healthy group, which was constrained by challenges in obtaining BAL samples. Nonetheless, this study offers crucial preliminary findings that pave the way for future investigations with larger cohorts, including examining associations between T cell biomarkers and clinical parameters (such as lung function and time) to CLAD. In conclusion, our study highlights that the T cell profile in peripheral blood may not necessarily reflect the T cell profile in the lung. The results demonstrate that T cell subsets in BAL and blood are unique and can vary considerably among lung transplant recipients, while previous studies have associated differences in BAL T cells with complications in lung transplantation, such as acute rejection and deteriorating lung function.

## 4. Methods

### 4.1. Patient Demographics

Healthy controls (termed ‘Healthy’) were undergoing bronchoscopy for chronic cough without any cause found, or for upper airway stenosis. Transplant recipients were allocated to being CLAD-affected” (‘CLAD’) or unaffected by CLAD (‘No CLAD’). A full description of subject demographics is provided in Table 1. Transplant recipients received maintenance immunosuppression consisting of Tacrolimus, Mycophenolate Mofetil and Prednisone.

### 4.2. Ethics Approval

Approval for this study was granted by the Human Research Ethics Committee, Metro North Hospital and Health Services, the Prince Charles Hospital (Brisbane, Australia) (HREC/2018/QPCH/44293), and written informed consent was obtained from all subjects involved before sample collection.

### 4.3. Isolation of PBMCs from Peripheral PBMC

Approximately 20 mL of heparinized PBMC was drawn from post-lung transplantation patients for PBMC isolation. PBMCs were separated through Lymphoprep-density-gradient centrifugation. Isolated cells were either stained for flow cytometry or cryopreserved in freezing buffer containing 35% fetal calf serum (FCS) (Gibco, Thermo Fisher Scientific, Seventeen Mile Rocks, Australia) and 7.5% Dimethylsulfoxide (DMSO) (Sigma, Merck life Science, Bayswater, Australia) for future analysis.

### 4.4. Isolation of Immune Cells from BAL

We promptly cooled 30–50 mL of BAL fluid on ice, followed by centrifugation at 1000× *g* for 10 min at 4 °C. Cell pellets were then resuspended in 1 mL of media (10% FCS in RPMI). These cells were subjected to flow cytometry immediately or cryopreserved in freezing buffer with a final concentration of 35% FCS and 7.5% DMSO for future analysis.

### 4.5. Flow Cytometric Analysis

Frozen BAL or PBMC samples were thawed and centrifuged, and the cell pellet was resuspended and washed with phosphate-buffered saline (PBS) (Gibco). Cells were stained with the antibodies listed in Table 2 Dead cells were excluded using Fixed Viability Stain 620 (BD Biosciences, Macquarie Park, NSW), and Fc receptor was blocked with TruStain FcX (BioLegend, San Diego, CA, USA). Intracellular staining for FoxP3, granzyme B, and Ki67 was carried out using the Transcription Buffer Set (BD Biosciences). Flow cytometric experiments were conducted using the BD LSRFortessa™ X-20 Cell Analyzer (BD Biosciences) and analyzed using KALUZA flow cytometry analysis software version 2.1 (Beckman Coulter, Brea, CA, USA). The gating strategy for identifying T cell populations is illustrated in Figure 1 for both BAL and PBMC. Cell subsets were quantified as percentages, and absolute counts were calculated per 100 mL of BAL. Table 2 lists the markers included in the study.

### 4.6. Lung Function Parameters

As part of routine lung transplant patient surveillance at the time of sample collection, we obtained spirometric measures. These measures were used to stratify samples based on Chronic Lung Allograft Dysfunction (CLAD) status at the time of BAL. CLAD was defined as a “persistent irreversible drop in lung function measured as forced expiratory volume in one second (FEV1) or forced vital capacity (FVC) to 20% or more from the post-transplant baseline after exclusion of alternate causes for reduced graft function,” in accordance with the International Society for Heart and Lung Transplantation (ISHLT) guidelines [5].

### 4.7. Statistical Analysis

Flow cytometry data was visualized using KALUZA flow cytometry analysis software version 2.1 (Beckman Coulter) and expressed as a percentage relative to the parent population for both BAL and PBMC. Statistical analysis was performed using GraphPad Prism software (Version 9.1.1). Comparisons between cell populations in paired PBMC and BAL were conducted using the Mann–Whitney test due to the non-normal distribution of the data. Associations between PBMC and BAL cell populations were evaluated using the Spearman correlation test. Additionally, comparisons between PBMC and BAL cell populations were carried out using the Wilcoxon test.

## Figures and Tables

**Table 1:** Patient demographics for the study cohort.

	Transplant (n = 20)	Non-Transplant (n = 4)
Gender		
Male	10	3
Female	10	1
Initial disease condition for transplant		
Cystic Fibrosis	11	N/A
COPD	4	N/A
IPF	4	N/A
Histiocytosis	1	N/A
Type of transplant		
Bilateral	19	N/A
Heart and Lung	1	N/A
Time post-transplant (months)	52.83 (SEM ± 10.84)	N/A
Age at bronchoscopy (years)	43.66 (SEM ± 3.78)	46.96 (SEM ± 9.38)

N/A = not applicable.

**Table 2:** Markers used in the multi-colour flow cytometric panel.

Marker	Purpose	Antibody Used
CD3	Marker for T lymphocytes	anti-CD3-AF700 (BioLegend)
CD4	Marker for CD4 T cells	anti-CD4-BV510 (BioLegend)
CD8	Marker for CD8 T cells	anti-CD8-APC-H7 (BD Biosciences)
CD14	Marker of macrophages and granulocytes.	anti-CD14-PE-Cy 7 (BD Biosciences)
CD25	Regulatory T cells express high CD25	anti-CD25-BV421 (BD Biosciences)
CD127	Regulatory T cells express low CD127	anti-CD127-FITC (BD Biosciences)
CD39	Immunosuppressive ecto-enzyme converts ATP to ADP	anti-CD39-BV711 (BioLegend)
CD73	Immunosuppressive ecto-enzyme coverts ADP to AMP	anti-CD73-BV605 (BD Biosciences)
CD194	Marker of cell migration, also known as CCR4	anti-CD194-PE-Cy 7 (BD Biosciences)
CD45RO	Marker of memory cells	anti-CD45RO-BV650 (BD Biosciences)
HLA-DR	Marker of MHC II and upregulated in activated Treg	anti-HLA-DR-BV785 (BioLegend)
Ki67	Marker of cellular proliferation	anti-Ki67-PerCp- Cy 5.5 (BioLegend)
FOXP3	Transcription factor specific to regulatory T cells	anti-FoxP3-PE (BD Biosciences)
Granzyme B	Serine protease commonly found in the granules of cytotoxic T cells	anti-granzyme B-APC (Invitrogen)

## Data Availability

The data that support the findings of this study are available on request from the corresponding author.
